# Complete Resolution of Unprovoked Left Renal Vein Thrombosis and Pulmonary Embolism Associated With Severe Iron Deficiency Anemia: A Five-Year Follow-Up Case Report and Dual-Energy CT Appraisal

**DOI:** 10.7759/cureus.115802

**Published:** 2026-09-04

**Authors:** Yousef H Hindi, Madona Akhobadze

**Affiliations:** 1 Medicine, New Vision University, Tbilisi, GEO; 2 Neuroscience, New Vision University, Tbilisi, GEO; 3 Neurosurgery, Georgian-Dutch Hospital, Tbilisi, GEO

**Keywords:** dual-energy ct, iron deficiency anemia, pulmonary embolism, renal vein thrombosis, rivaroxaban, vascular recanalization

## Abstract

Combined renal vein thrombosis (RVT) and pulmonary embolism (PE) in young adults without nephrotic syndrome, malignancy, or an identified major thrombophilia is uncommon. Severe iron deficiency anemia (IDA) has been associated with increased thrombotic risk, although a causal relationship cannot be established from an individual case. A 21-year-old male patient presented with flank and chest pain. Diagnostic workup confirmed acute left RVT and an acute embolism of the posterior basal segmental artery of the right lower lobe with pulmonary infarction, concurrent with profound microcytic IDA (hemoglobin 5.7 g/dL, MCV 58 fL) attributed clinically to severe chronic active gastritis, with concomitant vitamin B12 and folate deficiency and reactive thrombocytosis. Available thrombophilia testing was negative, including Factor V Leiden mutation and anticardiolipin IgG/IgM antibodies. The patient was treated with intravenous iron, blood transfusions, therapeutic low-molecular-weight heparin, and subsequently rivaroxaban 20 mg daily. At five-year follow-up, dual-energy CT (DECT) demonstrated complete anatomical recanalization of the left renal vein, complete resolution of the PE, and uniform pulmonary perfusion without residual segmental perfusion defects. Prior reports linking IDA to unusual-site venous thromboembolism (VTE) have relied largely on short-term clinical follow-up. This case contributes structural and functional imaging confirmation obtained five years after the index event, providing longitudinal anatomical and perfusion evidence of complete recanalization that is not available in the existing IDA-VTE literature. This case illustrates an unusual combination of RVT, PE, and profound IDA in a young adult without an identified conventional provoking factor. Severe IDA, occurring alongside broader micronutrient deficiency, may have contributed to thrombotic risk in this patient, but causality cannot be established. Complete vascular recanalization and clinical stability were observed during long-term anticoagulation with rivaroxaban and correction of the hematologic abnormalities.

## Introduction

Renal vein thrombosis (RVT) is a rare form of venous thromboembolism (VTE), most commonly associated with nephrotic syndrome, renal malignancy, trauma, or other prothrombotic conditions [1,2]. CT angiography is an established diagnostic modality, while anticoagulation is the principal treatment in most adult presentations [1-3]. Concomitant RVT and pulmonary embolism (PE) have been described but remain uncommon, particularly when an obvious provoking condition is absent [4,5]. PE classically arises from a combination of venous stasis, endothelial injury, and hypercoagulability, with recognized risk factors including immobilization, recent surgery or trauma, malignancy, and inherited or acquired thrombophilias. Clinical presentation is highly variable, ranging from asymptomatic disease to dyspnea, pleuritic chest pain, tachycardia, and hemodynamic compromise, and can be atypical or subtle in younger patients without conventional risk factors, as in the case described here [6]. Factor V Leiden, the most common inherited thrombophilia, results from a point mutation in the F5 gene that renders factor V resistant to inactivation by activated protein C. It is inherited in an autosomal dominant pattern, and approximately 3%-8% of individuals of European descent carry the heterozygous form, with homozygotes at substantially greater thrombotic risk [7]. A family history of Factor V Leiden, as noted in this patient's relatives, raises the possibility of an inherited contribution to thrombotic risk even when the patient's own testing is negative, particularly given the limitations of functional assay testing discussed later in this report.

Severe iron deficiency anemia (IDA) has been associated with arterial and venous thrombosis in case reports and observational studies. Proposed mechanisms include reactive thrombocytosis, altered erythrocyte rheology, and changes associated with iron-restricted erythropoiesis [8-10]. A recent multi-institutional retrospective study also reported an association between IDA and VTE, which was attenuated after excluding patients who developed thrombocytosis [10]. These data support consideration of IDA as a potential contributor to thrombotic risk, but they do not establish causality in an individual case.

Existing IDA-VTE case reports have generally documented the acute presentation and short-interval clinical or laboratory recovery, without long-term structural or functional vascular imaging. We present a five-year follow-up case of a young male patient with concurrent RVT, PE with pulmonary infarction, and severe IDA, with complete anatomical recanalization and normal follow-up pulmonary perfusion on dual-energy CT (DECT). Imaging follow-up at this duration and modality is not, to our knowledge, previously reported in this clinical context.

## Case presentation

Initial presentation and diagnostic evaluation (August 2018)

An otherwise healthy 21-year-old male patient presented with severe left flank and chest pain. Contrast-enhanced CT of the chest and abdomen, performed on a Philips IQon Spectral CT scanner (Hadassah University Medical Center, Ein Kerem, Jerusalem, Israel), revealed an acute left RVT alongside an acute embolism of the posterior basal segmental artery of the right lower lobe, complicated by pulmonary infarction.

Laboratory investigations revealed severe microcytic IDA, with a hemoglobin level of 5.7 g/dL (reference range: 13.5-17.5 g/dL), mean corpuscular volume (MCV) of 58 fL (reference range: 80-100 fL), and low serum iron of 36.2 µg/dL (reference range: 60-170 µg/dL). Peripheral blood smear demonstrated marked hypochromia, microcytosis, anisocytosis, and target cells. Reactive thrombocytosis was present, with a platelet count of up to 630 × 10⁹/L (reference range: 150-400 × 10⁹/L), along with leukocytosis of 13 × 10⁹/L (reference range: 4-11 × 10⁹/L). There was no laboratory evidence of hemolysis. During the admission, the patient was diagnosed with vitamin B12 and folate deficiencies and received supplementation; folic acid deficiency was specifically documented in September 2018. Bone marrow biopsy demonstrated normal hematopoiesis without evidence of malignancy or myelodysplasia.

Gastrointestinal evaluation, including esophagogastroduodenoscopy, revealed severe chronic active gastritis without evidence of active gastrointestinal hemorrhage. *Helicobacter pylori* immunostaining was negative. The patient had no gastrointestinal symptoms suggestive of celiac disease, and celiac serology was not performed. Available thrombophilia testing was unrevealing, including testing for the Factor V Leiden mutation and antiphospholipid antibodies (anticardiolipin IgG and IgM). Family history was notable for a relative with deep vein thrombosis and another with the Factor V Leiden mutation.

Initial management and intermediate course

The patient received packed red blood cell transfusions and intravenous iron sucrose (Venofer), resulting in prompt hematologic recovery. Anticoagulation was initiated with therapeutic low-molecular-weight heparin (enoxaparin 60 mg twice daily with monitored anti-Xa levels) and transitioned to oral rivaroxaban 20 mg once daily.

Three months later (November 2018), the patient presented with localized left chest pain following strenuous swimming. Physical examination and vital signs were within normal limits (blood pressure 143/65 mmHg, heart rate 68 bpm, SpO2 97% on room air). Laboratory parameters demonstrated full hematologic recovery and normal inflammatory and cardiac markers (Table 1). The chest pain was diagnosed as self-limiting musculoskeletal strain, and rivaroxaban therapy was continued.

**Table 1:** Laboratory parameters during follow-up (November 2018)

Parameter	Value	Reference range
Hemoglobin	15.1 g/dL	13.5-17.5 g/dL
High-sensitivity troponin T	0.013 ng/mL	<0.014 ng/mL
D-dimer	0.10 μg/mL	<0.50 μg/mL (FEU)
C-reactive protein	<0.5 mg/dL	<0.5 mg/dL
Serum creatinine	85 μmol/L	62-106 μmol/L
Blood urea	4.0 mmol/L	2.5-7.1 mmol/L

Five-year long-term follow-up and radiological confirmation (2023-2024)

In November 2023, a comprehensive follow-up evaluation utilizing DECT of the chest, abdomen, and pelvis was performed at Hadassah University Medical Center, Ein Kerem, Jerusalem, Israel, using a Philips IQon Spectral CT scanner to assess long-term vascular recanalization. The findings are summarized in Table 2.

**Table 2:** Comparison of baseline (2018) and five-year follow-up (2023) imaging DECT-PBV: dual-energy CT pulmonary blood volume.

Anatomical region	Baseline CT (August 2018)	Follow-up DECT (November 2023)
Left renal vein	Acute occlusive thrombus	Complete recanalization
Posterior basal segmental artery, right lower lobe	Acute embolism with infarction	Complete resolution
Pulmonary perfusion (DECT-PBV)	Segmental perfusion defect	Uniform perfusion, with no defect
Spleen size	Enlarged (13.9 cm)	Normalized (12.1 cm)

Abdominal DECT demonstrated complete patency and recanalization of the left renal vein with normal bilateral renal cortical enhancement (Figure 1). Chest DECT pulmonary vascular imaging demonstrated total resolution of the previous pulmonary embolism and resolution of the right lower lobe pulmonary infarction (Figure 2). Dual-energy pulmonary blood volume (DE-PBV) perfusion mapping demonstrated uniform pulmonary iodine distribution without residual segmental perfusion defects (Figures 3, 4).

**Figure 1:**
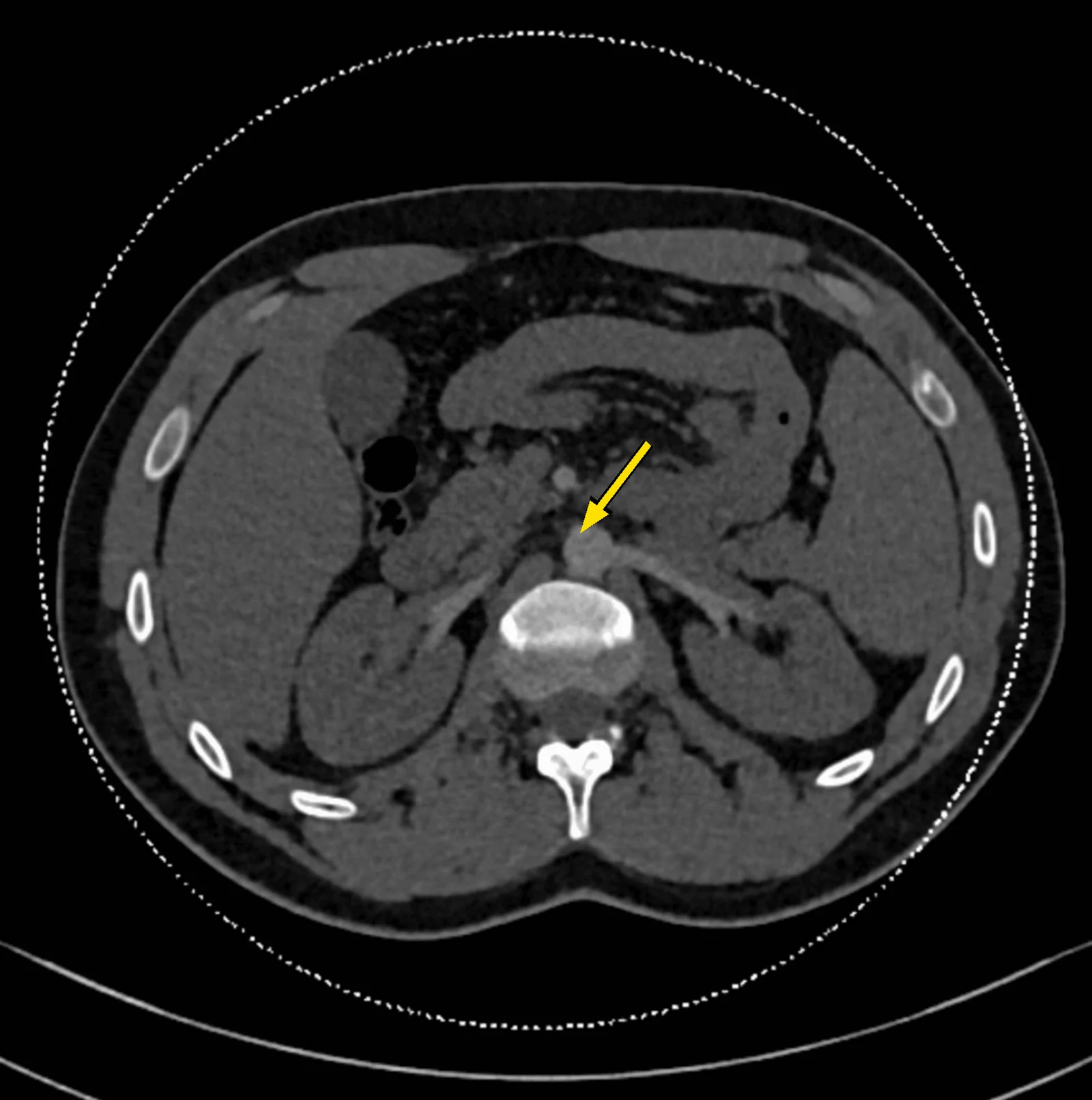
Axial contrast-enhanced abdominal CT, renal vein level (five-year follow-up; November 2023) The arrow indicates the left renal vein, which is patent with no residual filling defect, with symmetric bilateral renal parenchymal enhancement.

**Figure 2:**
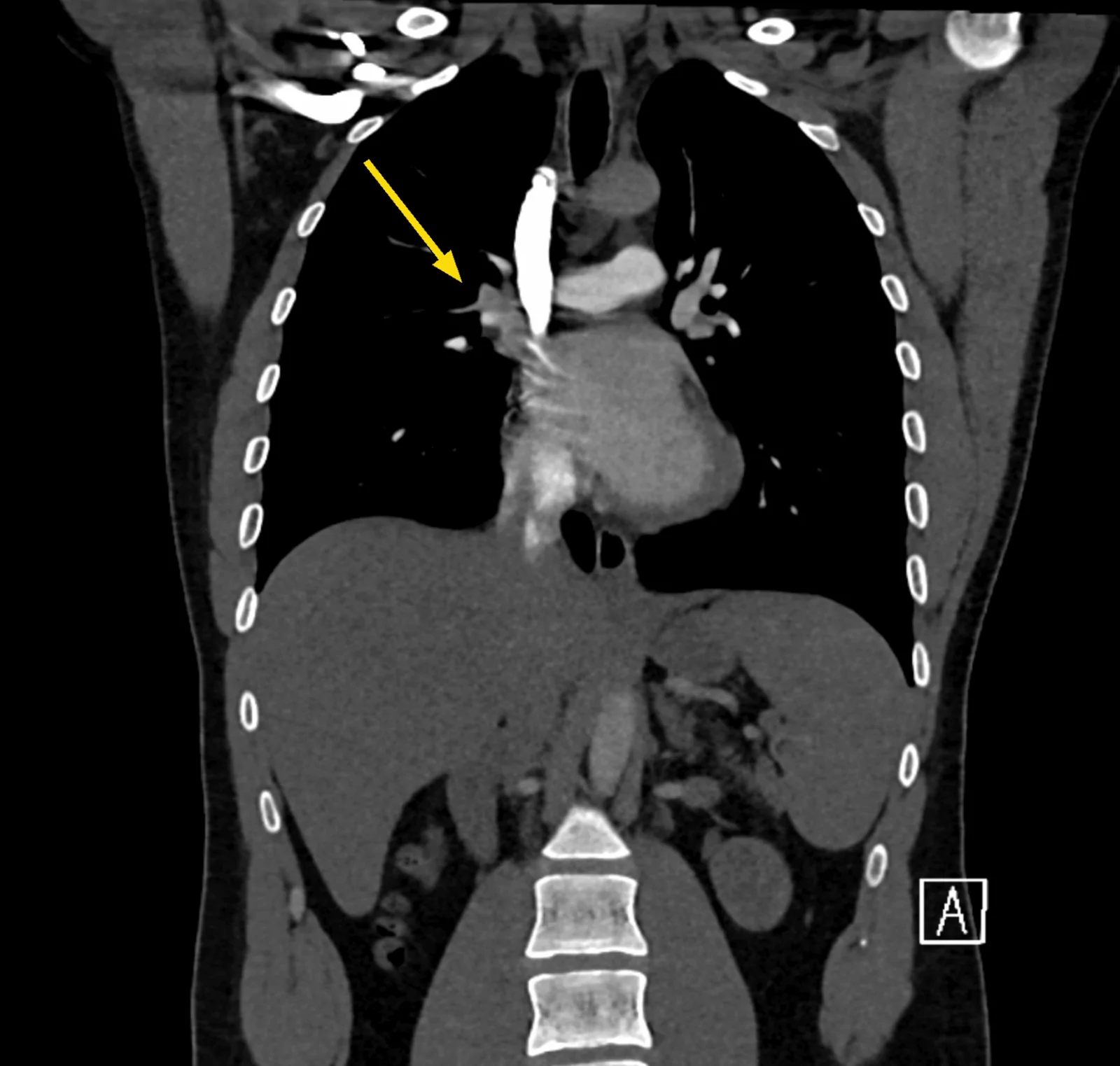
Coronal dual-energy CT of the chest (five-year follow-up) The arrow indicates the contrast-opacified central pulmonary vasculature, widely patent with no filling defect.

**Figure 3:**
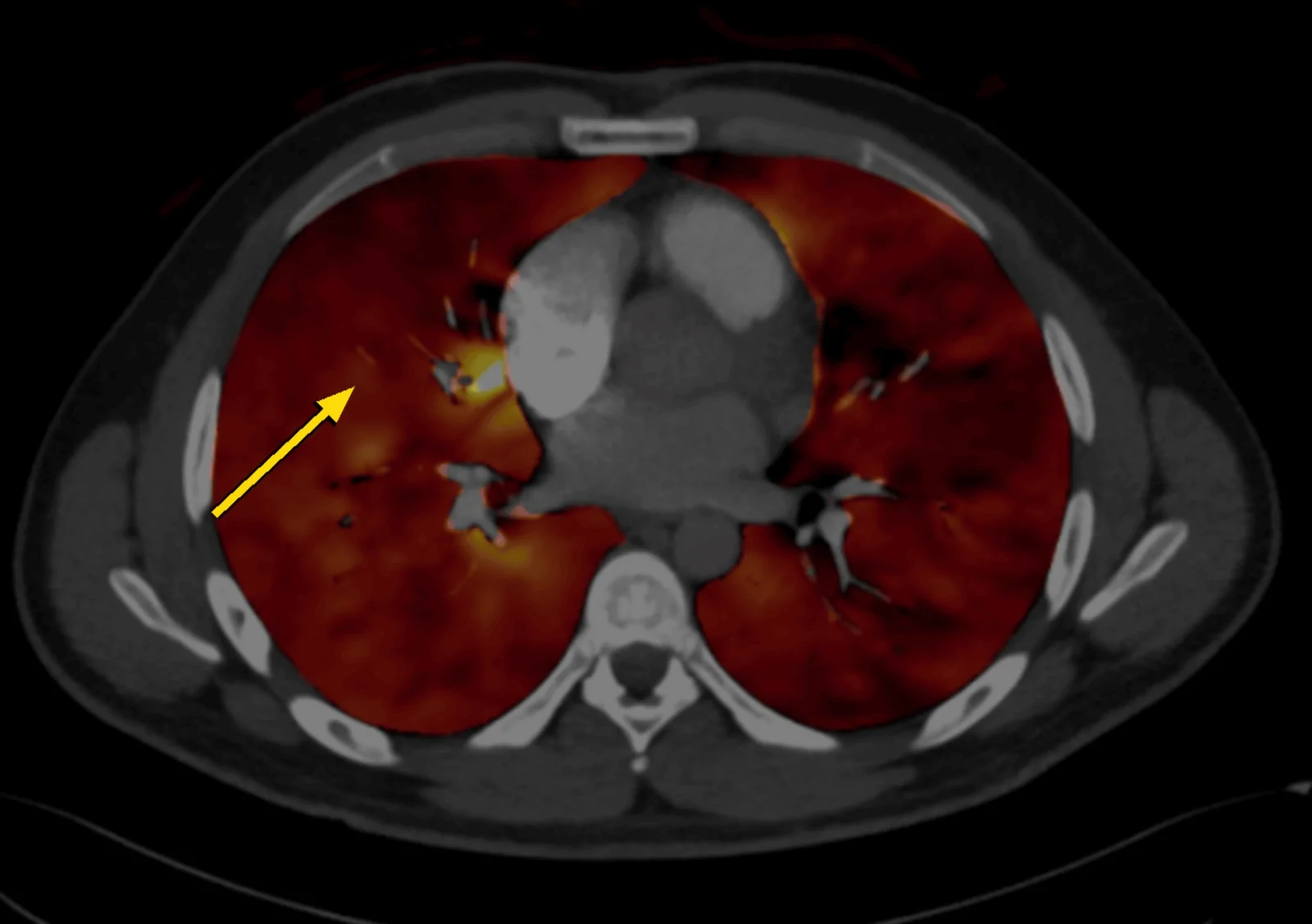
Dual-energy CT pulmonary blood volume (DE-PBV) axial perfusion map The arrow indicates homogeneous iodine (perfusion) signal in the posterior basal segment of the right lower lobe, confirming resolution of the prior segmental defect.

**Figure 4:**
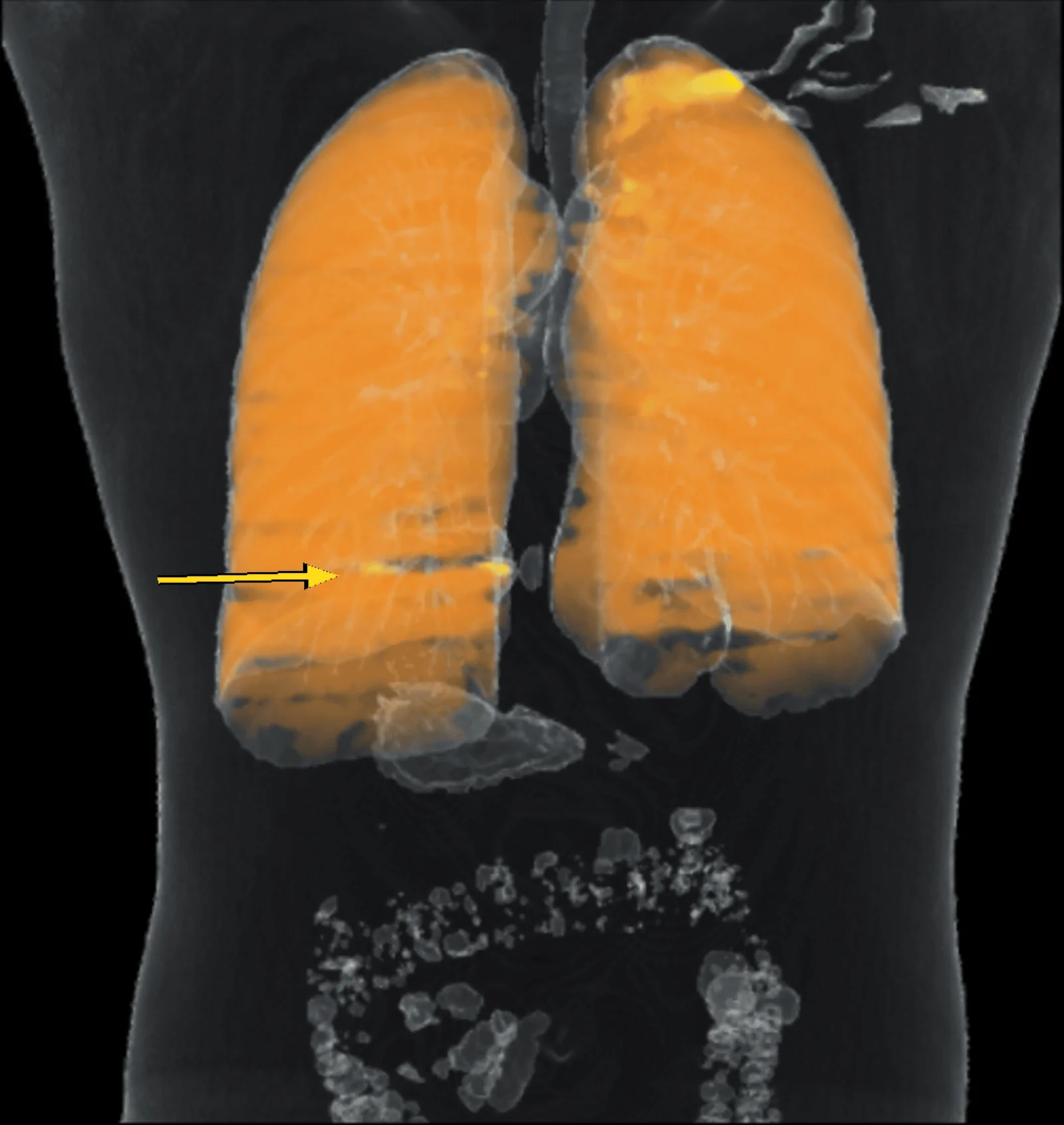
Coronal 3D volume-rendered dual-energy CT lung perfusion reconstruction The arrow indicates the posterior basal segment of the right lower lobe with full, uniform perfusion and no residual defect.

Incidental abdominal findings included a stable 15 x 5 mm splenic hemangioma, spleen size normalization from 13.9 cm to 12.1 cm, and a small left inguinal hernia containing fat stranding. Laboratory evaluation confirmed normal serum lactate dehydrogenase (LDH 179 U/L) and negative urine cultures. Follow-up lung-window imaging confirmed clear right lower lobe parenchyma without residual consolidation or scarring (Figure 5). Outpatient hematology follow-up in February 2024 confirmed persistent clinical remission, defined by the complete absence of recurrent VTE symptoms alongside the previously documented imaging and laboratory resolution.

**Figure 5:**
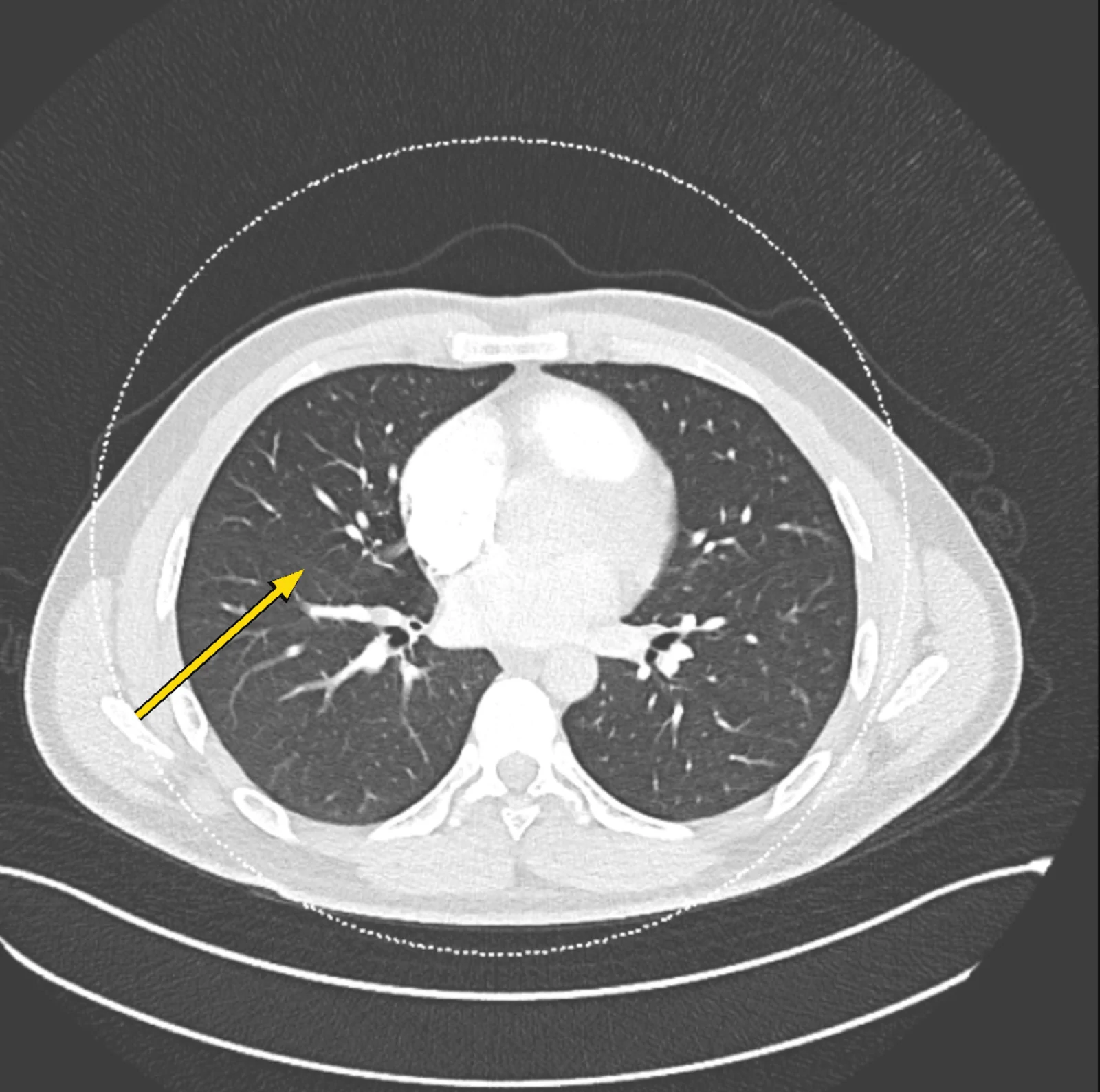
Axial lung-window CT at follow-up The arrow indicates the right lower lobe, which is clear with no residual consolidation or scarring.

## Discussion

This case highlights two clinical considerations: the possible contribution of profound IDA, occurring within a broader picture of combined micronutrient deficiency, to an otherwise unexplained VTE presentation, and the use of DECT to document long-term anatomical and perfusion findings after PE.

Potential contribution of severe IDA to VTE

The relationship between IDA and thrombosis has been described in clinical reports and mechanistic studies, with proposed pathways involving reactive thrombocytosis, altered erythrocyte rheology, and iron-restricted erythropoiesis [8-10]. In this patient, the profound anemia (hemoglobin 5.7 g/dL, MCV 58 fL) occurred concurrently with marked reactive thrombocytosis (platelet count up to 630 ×10⁹/L), RVT, and PE. Iron deficiency is a recognized cause of reactive thrombocytosis, thought to result from increased thrombopoietin sensitivity and enhanced megakaryocyte stimulation during iron-restricted erythropoiesis [8-10]. This reactive thrombocytosis, together with altered erythrocyte rheology, has been proposed as a contributor to thrombotic risk distinct from clonal thrombocytosis seen in myeloproliferative disease. The temporal association and subsequent hematologic correction raise the possibility that severe IDA contributed to thrombotic susceptibility, but this case cannot establish that IDA was the cause of the thrombotic events.

This patient's anemia was not isolated iron deficiency: concomitant vitamin B12 and folate deficiency were also documented at presentation. This combined micronutrient deficiency pattern is relevant to the causal narrative in two ways. First, it raises the possibility of an underlying gastrointestinal process, with severe chronic active gastritis identified on EGD (*H. pylori* immunostaining negative), contributing to the combined nutritional deficiency state. Malabsorptive states have themselves been proposed as independent contributors to thrombotic risk through mechanisms including hyperhomocysteinemia (from B12/folate deficiency) and altered coagulation factor and rheological status [11]. Second, it means the mechanistic pathways specific to iron-restricted erythropoiesis and reactive thrombocytosis cannot be cleanly isolated from those attributable to B12/folate-related hyperhomocysteinemia or to malabsorption more broadly. We therefore frame this case as demonstrating a combined nutritional-deficiency state associated with VTE, rather than attributing thrombotic risk to iron deficiency in isolation; iron deficiency is highlighted because it was the most severe and clinically dominant abnormality, not because it was mechanistically isolated from the other deficiencies.

Prior reports linking IDA to unusual-site VTE have relied largely on short-term clinical follow-up. This case contributes structural and functional imaging confirmation, dual-energy CT with iodine-based pulmonary blood volume mapping, obtained five years after the index event, providing longitudinal anatomical and perfusion assessment at five years, an imaging follow-up interval and modality that, to our knowledge, have not been previously reported in this clinical context.

Rivaroxaban in unusual-site venous thrombosis

In this case, anticoagulation began with therapeutic low-molecular-weight heparin and was subsequently continued with rivaroxaban 20 mg once daily. Given the unprovoked nature of the combined RVT and PE, anticoagulation was continued long-term under hematology follow-up; the rivaroxaban dose was later reduced to 10 mg once daily per treating hematology guidance, and the patient remained on anticoagulant therapy at last follow-up, with no specific discontinuation date planned. Complete anatomical recanalization of the left renal vein and resolution of the pulmonary embolism were observed at five-year follow-up. These findings describe a favorable individual outcome during rivaroxaban therapy; they do not, by themselves, establish the efficacy or superiority of rivaroxaban for this indication [12,13].

Utility of dual-energy CT perfusion imaging

DECT can provide pulmonary perfusion information complementary to conventional CT pulmonary angiography by generating iodine-based pulmonary blood volume maps [14-16]. In this patient, follow-up DECT demonstrated patent pulmonary arteries and uniform pulmonary perfusion without residual segmental defects. These findings support the absence of detectable residual perfusion abnormality at the time of follow-up. DECT findings alone, however, should not be interpreted as proof that chronic thromboembolic pulmonary hypertension was prevented or definitively excluded.

Incidental splenic findings

Follow-up imaging showed spleen size reduction from 13.9 cm at baseline to 12.1 cm at five years, alongside a stable 15 x 5 mm splenic hemangioma. The baseline splenomegaly may have reflected a reactive or hemodynamic process occurring in the setting of severe anemia; congestive splenomegaly related to renal vein thrombosis is also a plausible contributor, although the available imaging cannot distinguish between these possibilities, with normalization occurring alongside hematologic recovery. Portal hypertension, myeloproliferative disease, and lymphoproliferative or infiltrative disease were not otherwise supported by this patient's normal LDH, normal bone marrow biopsy, and absence of other systemic findings, making these considerably less likely explanations. The persistent, stable hemangioma is considered an incidental finding unrelated to the thrombotic or hematologic presentation.

Limitations

This report is subject to several limitations inherent to single-patient case reporting. First, and most fundamentally, no causal relationship can be established between IDA (or the broader micronutrient deficiency state) and this patient's thrombotic events from a single case; the temporal association observed here is compatible with, but does not prove, a contributory role. Second, the anemia was multifactorial (iron, B12, and folate deficiency in the setting of chronic gastritis), which precludes attributing thrombotic risk to any single deficiency in isolation. Third, other unmeasured or incompletely characterized prothrombotic factors cannot be excluded; the patient's family history of Factor V Leiden mutation and venous thromboembolism in relatives, despite negative Factor V Leiden testing in the patient himself, raises the possibility of an unidentified heritable contributor to thrombotic risk. Factor V Leiden testing was performed using a functional plasma-based assay (activated protein C resistance) rather than direct genetic testing, and blood was drawn after the patient had already received red blood cell transfusion. Because this assay reflects plasma clotting factor activity rather than genotype, transfusion and the acute prothrombotic/inflammatory state could theoretically have influenced the result, and a negative result obtained under these conditions carries more uncertainty than a genotype-confirmed negative would; confirmatory genetic testing was not performed. A single antiphospholipid antibody measurement during acute illness is likewise not sufficient to exclude antiphospholipid syndrome, which requires confirmatory testing at least 12 weeks later; repeat testing was not performed in this patient. Fourth, portions of the 2018 index presentation are reconstructed retrospectively from the medical record rather than documented prospectively for research purposes, which carries the usual limitations of retrospective clinical documentation. Finally, as an observation from a single patient, this report cannot support generalizable conclusions about the efficacy of rivaroxaban in RVT or about the expected natural history of DECT perfusion findings after unusual-site VTE. It is intended to describe a clinical observation and generate hypotheses for future systematic study, not to establish causal or therapeutic claims.

## Conclusions

This case illustrates an unusual combination of left RVT, PE, and profound IDA occurring within a broader combined micronutrient deficiency state, in a young adult without an identified conventional provoking factor, though a family history of Factor V Leiden mutation and venous thromboembolism in relatives raises the possibility of an unidentified heritable contributor. Severe IDA, marked by pronounced microcytosis (MCV 58 fL) and reactive thrombocytosis (platelet count up to 630 ×10⁹/L), may have contributed to thrombotic risk in this patient, but a causal relationship cannot be established from a single case, and the contribution of concurrent B12/folate deficiency and the underlying gastrointestinal process (chronic gastritis) cannot be excluded; celiac disease was not specifically screened for, as the patient had no suggestive symptoms. Complete clinical recovery, renal vein recanalization, pulmonary embolus resolution, and normal follow-up pulmonary perfusion were observed during treatment with hematologic repletion and long-term rivaroxaban. Longitudinal dual-energy CT at five years provides structural and functional imaging confirmation of this outcome not previously reported in this clinical context.
